# Clinical In-Hospital Outcomes of Acute Myocardial Infarction in Patients With Hematological Malignancies

**DOI:** 10.7759/cureus.21627

**Published:** 2022-01-26

**Authors:** Muhammad Z Khan, Abdul Baqi, Kirtenkumar Patel, Joshua Weinstock, Sona Franklin, Steven Kutalek

**Affiliations:** 1 Cardiology, University Hospitals Cleveland Medical Center, Cleveland, USA; 2 Internal Medicine, St. Vincent Medical Center - Mercy Health, Toledo, USA; 3 Cardiology, North Shore University Hospital, New York, USA; 4 Cardiology, Cooper University Hospital, New Jersey, USA; 5 Internal Medicine, St. Mary Medical Center, Langhorne, USA; 6 Cardiology, St. Mary Medical Center, Langhorne, USA

**Keywords:** cost, length of stay, mortality, hematological malignancies, acute myocardial infarction

## Abstract

Introduction

The purpose of our study is to determine in-hospital outcomes of acute myocardial infarction in patients with hematological malignancies and their subtypes.

Method

Patient data were obtained from the nationwide inpatient sample (NIS) database between the years 2009-2014. Patients with hematological cancer subtypes and acute MI (non-ST segment elevation myocardial infarction and ST-segment elevation myocardial infarction (NSTEMI/STEMI) were identified using validated international classification of diseases (ninth revision) and clinical modification (ICD-9-CM) codes. Statistical analysis using the chi-square test was performed to determine the hospital outcomes of acute MI in patients with hematological cancers and subtypes.

Results

The prevalence of acute myocardial infarction was 2.4% in patients with hematological neoplasms (N=3,027,800). Amongst the subtypes of blood cancers, the highest prevalence of acute MI was seen in lymphocytic leukemia (2.9%). The mortality of MI in patients with hematological malignancies was 16.8% vs 8.8% in patients with non-hematological malignancies, in-hospital costs were $25469 ± 36763 vs. $20534 ± 24767, and length of in-hospital stay was 8.3 ± 10 vs 6.3 ± 7.8 days. Amongst the hematological cancer subtypes, the highest mortality of acute MI was found in myeloid leukemia (23%) followed by multiple myeloma (MM) (17.9%), lymphocytic leukemia (15.9%), and lymphoma (14.4%). The length of stay and hospitalization cost was highest for myeloid leukemia, followed by MM, lymphocytic leukemia, and lymphoma.

Conclusion

This study showed that acute MI in patients with hematological malignancies has higher in-hospital mortality, length of stay, and cost. Amongst the blood neoplasm subtypes the highest mortality, length of hospital stay, and hospitalization cost were found in myeloid leukemia.

## Introduction

Cancer and acute coronary syndrome (ACS) are the leading causes of morbidity and mortality in the United States. The prevalence of myocardial infarction (MI) is high in cancer patients due to complex pathophysiology. The risk of myocardial infarction not only depends on the traditional risk factors of coronary artery disease but also cancer type, stage, treatment, and associated hypercoagulopathy [1]. There is limited data regarding the incidence of MI (non-ST segment elevation myocardial infarction and ST-segment elevation myocardial infarction) in hematological malignancies. Li et al. reported that hematological malignancies cause thrombosis due to hyperviscosity and or leukemic clots, which can cause decreased blood flow through the coronary arteries. Management of clots due to hyperviscosity in MI patients is different from atherosclerotic clots. Blood flow in MI is usually restored after transcatheter aspiration in hyperviscosity clots compared to stenting or grafts for reperfusion in atherosclerotic CAD [2]. As a result, in-patient management, mortality, length of stay, and cost can vary in MI due to hyperviscosity or leukemic clots.

Data regarding mortality of acute myocardial infarction in patients with underlying hematological neoplasms is limited. Our study aims to identify the inpatient mortality of MI in hematological neoplasm and their subtypes. Our secondary outcomes include determining the inpatient hospital stay and cost of MI in patients with hematological neoplasm.

## Materials and methods

Data sources

The present study was conducted using the National Inpatient Sample (NIS) database, the largest inpatient database in the United States. The NIS data were collected from 48 states, representing more than 97% of the US population and 7-8 million discharges each year. NIS data is obtained from more than 7 million hospital stays each year, and it estimates more than 35 million hospitalizations nationally. Each admission contains information on patient characteristics including demographics, comorbidities, complications, and primary and secondary discharge diagnoses. This has been explained in detail in previous studies [3,4]. The International Classification of Disease, 9th revision, Clinical Modification (ICD 9-CM) codes were used to identify diagnosis in the NIS database [5,6]. Data included in this study were obtained between January 2009 and December 2014. NIS data includes the charge-to-cost ratio. Charges showed the hospital bills for services while cost represents how much the service costs, including utility cost, supplies, and wages. All deaths from January 2009 to December 2014 are reported as mortality of patients with acute MI. The study cohort was derived from a de-identified and publicly available database; hence, the study was considered exempt from the formal approval of the institutional review board. We used personal money to buy this data. We did not use any source of funding.

Diagnosis codes for MI and hematological cancer

CCS (Clinical Classifications Software, Healthcare Cost and Utilization Project, Agency for Healthcare Research and Quality, Rockville, MD) codes for nonspecific and specific malignant cancers were used to extract specific cancers included in our study as can be seen in the appendix. The NIS data provides up to 30 CCS diagnoses for each inpatient visit.

We extracted myocardial infarction (MI) and cancer hospitalizations using appropriate ICD-9-CM diagnosis codes in primary or secondary diagnosis (see table in the appendix). Furthermore, we documented the following comorbidities: hypertension, diabetes, obesity, and chronic kidney disease (CKD) in our study cohort. The present study included Hodgkin's lymphoma, multiple myeloma, non-Hodgkin's lymphoma (NHL), and leukemia. CCS codes from 11 to 43 were used for extraction of specific cancers which were included in our study. In our study, acute MI is the combination of NSTEMI and STEMI populations.

Statistical analysis

The data analysis and extraction were done using SAS statistical software version 9.4. All continuous variables, such as length of stay, and hospitalization cost, were compared using Student's t-test. These variables were presented as a mean and standard deviation (SD) for normally distributed variables, while median and interquartile ranges were used for non-Gaussian distributed variables. On the other hand, categorical variables such as in-hospital mortality were analysed using the Pearson chi-square test. These variables were presented as a weighted frequency in percentages. A p-value <0.05 was considered statistically significant.

## Results

**Figure 1:**
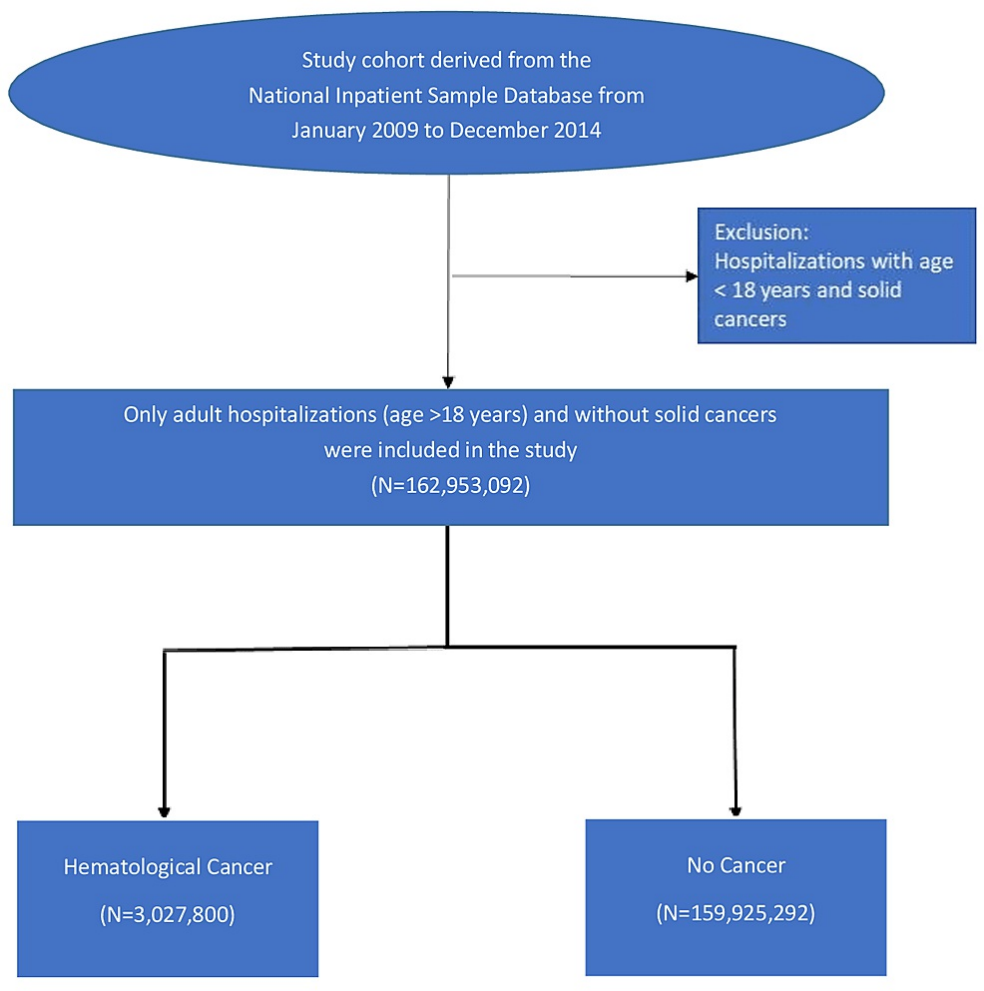
Flow chart Flow chart of the study selection process

Between the years 2009 and 2014, a total number of 288,727,118 adult hospitalizations were reported. After excluding patients with solid cancers and those who were < 18 years, 162,953,092 were included in the study (Figure 1).

The incidence of acute MI in haematological cancer patients was 2.4% (Table 1).

**Table 1:** Patient-level characteristics Patient-level characteristics of hematological cancer vs no cancer in 2009-2014.

	Hematologic cancer	No cancer	P value
Clinical Characteristics			
N=162,953,072	N=3,027,800 (1.9%)	N=159,925,292 (98.1%)	
Gender			< .0001
Male (%)	54.7	39.4	
Female (%)	45.3	60.6	
*missing data (N=107,665)			
Race			< .0001
Caucasians (%)	68	61.3	
African Americans (%)	10.7	13.9	
Other race (%)	21.3	24.7	
*missing data (N=6562)			
Comorbidity			
Obstructive sleep apnea (%)	4.1	4.7	< .0001
Hyperlipidemia (%)	22.5	22.4	< .0001
Chronic pulmonary disease (%)	16.6	17.4	< .0001
Hypertension (%)	48.9	47.1	< .0001
Diabetes mellitus (%)	23.1	22.9	< .0001
Renal failure (%)	15.6	11.3	< .0001
Obesity (%)	7.3	11.6	< .0001
Alcohol abuse (%)	1.7	4.9	< .0001
Drug abuse (%)	1.8	4.7	< .0001
Rheumatoid arthritis/collagen vascular disease (%)	2.9	2.6	< .0001
Acute myocardial infarction (%)	2.4	3.1	< .0001

Amongst the blood neoplasms subtypes, the highest incidence of ACS was found in lymphocytic leukemia followed by multiple myeloma, lymphoma, and myelocytic leukemia (Table 2).

**Table 2:** Different types of hematological cancer Patient-level characteristics of different types of hematological cancer vs no cancer in 2009-2014. HLD=hyperlipidemia, HTN=hypertension, MI=myocardial infarction, MM=multiple myeloma, RA=rheumatoid arthritis

	Lymphocytic leukemia (N= 533,878; 0.3%)	No cancer (N=159,925,292; 98.1%)	P value	Myeloid leukemia (N=462,042; 0.3%)	No cancer (N=159,925,292; 98.1%)	P value	Lymphoma (N=1,447,807; 0.9%)	No cancer (N=159,925,292;98.1%)	P value	MM (N=515,747; 0.3%)	No cancer (N=159,925,292; 98.1%)	P value
Clinical Characteristics												
Gender			< .0001			< .0001			< .0001			< .0001
Male (%)	57.5	39.6		54.7	39.6		54.3	39.5		53.4	39.6	
Female (%)	42.5	60.4		45.3	60.4		45.7	60.5		46.5	60.4	
*missing data (N=107665)												
Race			< .0001			< .0001			< .0001			< .0001
Caucasians (%)	71.2	61.4		68.4	61.4		69.4	61.4		59.9	61.5	
African Americans (%)	7.8	13.9		9.3	13.9		8.9	13.9		20.3	13.9	
Other race (%)	21	24.6		22.3	24.6		21.6	24.6		19.7	24.6	
*missing data (N=6562)												
Comorbidity												
Obstructive sleep apnea (%)	4.2	4.6	< .0001	4.2	4.6	< .0001	3.9	4.7	< .0001	4.2	4.6	< .0001
HLD (%)	24.4	22.4	< .0001	19.9	22.4	< .0001	22.6	22.4	< .0001	22.9	22.4	< .0001
Chronic pulmonary disease (%)	18.8	17.4	< .0001	14.9	17.4	< .0001	16.3	17.4	< .0001	16.3	17.4	< .0001
HTN (%)	51.3	47.2	< .0001	46.1	47.2	< .0001	47.7	47.1	< .0001	57.6	47.1	< .0001
Diabetes mellitus (%)	25	22.9	< .0001	21.9	22.9	< .0001	22	22.9	< .0001	25	22.9	< .0001
Renal failure (%)	14.8	11.4	< .0001	12.3	11.4	< .0001	11.9	11.4	< .0001	30.4	11.4	< .0001
Obesity (%)	6.9	11.5	< .0001	7.7	11.5	< .0001	7.4	11.5	< .0001	6.9	11.5	< .0001
Alcohol abuse (%)	1.4	4.9	< .0001	1.4	4.9	< .0001	1.9	4.9	< .0001	1.2	4.9	< .0001
Drug abuse (%)	1.3	4.7	< .0001	1.7	4.7	< .0001	2.2	4.7	< .0001	1.2	4.7	< .0001
RA/collagen vascular disease (%)	2.4	2.6	< .0001	2.7	2.6	0.05	3.4	2.6	< .0001	2.2	2.6	< .0001
Acute MI (%)	2.9	3.1	< .0001	2.1	3.1	< .0001	2.1	3.1	< .0001	2.6	3.1	< .0001

Comparison of hematological cancer and baseline characteristics in subgroups

Hospitalizations due to MI in patients with hematological cancers were likely to be male and Caucasian compared to those without cancer in all groups(Table 1). The patients with hematological cancers have a higher prevalence of risk factors for CAD such as hypertension, diabetes mellitus, drug abuse, alcohol abuse, renal failure, obesity, collagen vascular disease, and hyperlipidemia as compared to the patients without any underlying malignancy (p<0.001) (Table 1). However, the prevalence of obstructive sleep apnea and chronic pulmonary disease was higher in those patients who had acute MI without any underlying cancer as compared to those who had underlying hematological malignancy (p< 0.001) (Table 1).

Comparison of hospital outcomes of MI (Mortality, length of stay, cost) in subtypes of hematological neoplasms

The primary and secondary hospital outcomes of the study are shown in Table 3.

**Table 3:** Hospital outcomes of acute myocardial infarction with different types of hematological cancer Hospital outcomes of acute myocardial infarction (MI) with different types of hematological cancer vs MI with no cancer from years 2009 to 2014. Dollars=$, MM=multiple myeloma

	Acute MI with hematological cancer	Acute MI with no cancer	p-value	Acute MI Lymphocytic leukemia	Acute MI with no cancer	p-value	Acute MI Myeloid leukemia	Acute MI with no cancer	p-value	Acute MI lymphoma	Acute MI with no cancer	p-value	Acute MI MM	Acute MI with no cancer	p-value
Mortality	16.8%	8.8%	< .0001>	15.9%	8.9%	< .0001>	23.9%	8.9%	< .0001>	14.4%	8.9%	< .0001>	17.9%	8.9%	< .0001>
Hospitalization cost	25469 ± 36763$	20534 ± 24767$	< .0001>	24327 ± 34433$	20596 ± 24960$	< .0001>	36176 ± 59713$	20575 ± 24862$	< .0001>	24596 ± 33773$	20584 ± 24933$	< .0001>	24864 ± 31725$	20596 ± 24976$	< .0001>
Length of stay	8.3 ± 10 days	6.3 ± 7.8 days	< .0001>	8 ± 9 days	6.4 ± 7.8 days	< .0001>	11.4 ± 15.7 days	6.4 ± 7.8 days	< .0001>	7.6 ± 8.7 days	6.4 ± 7.8	< .0001>	8.9 ± 9.5 days	6.4 ± 7.8 days	< .0001>

**Figure 2:**
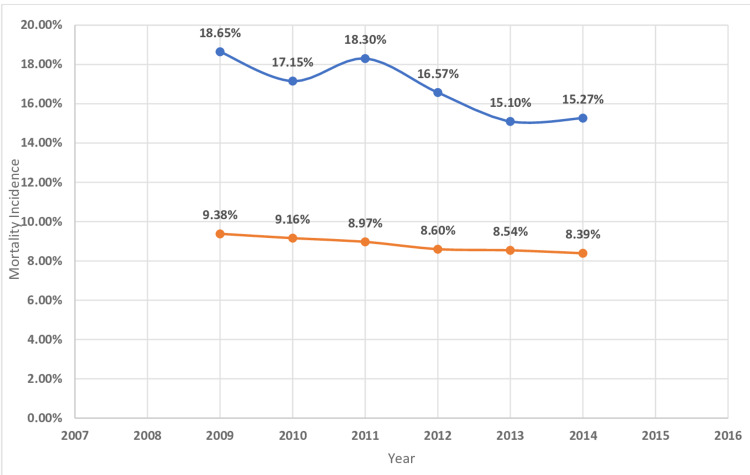
Mortality incidence in hematological cancer Trends in mortality incidence in hematological cancer with acute myocardial infarction. The blue curve indicates acute myocardial infarction with underlying hematological malignancy (%). The orange curve indicates acute myocardial infarction without underlying hematological malignancy (%).

**Figure 3:**
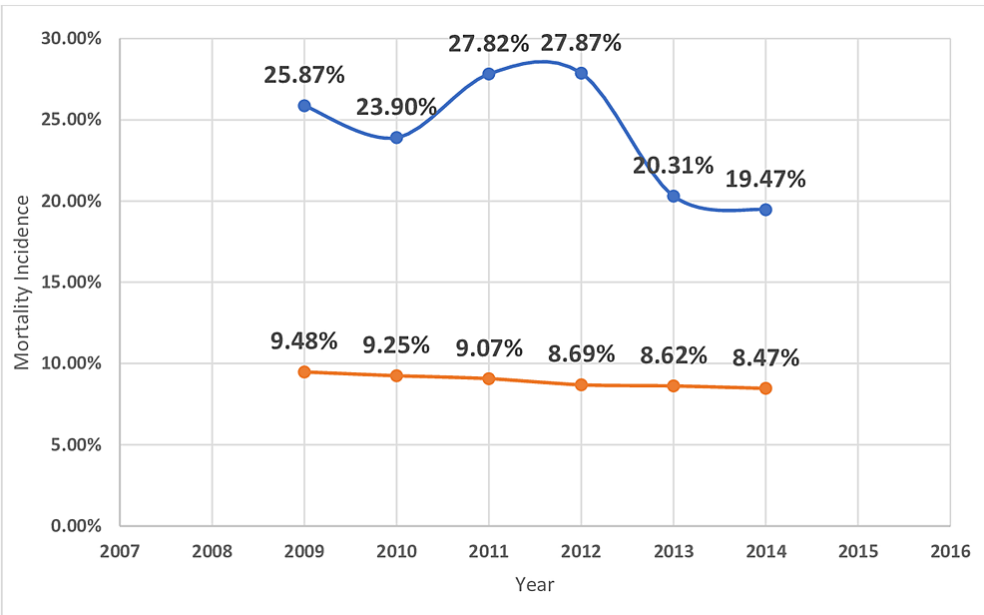
Mortality incidence in myeloid leukemia. Trends in mortality incidence in myeloid leukemia with acute myocardial infarction. The blue curve indicates myocardial infarction in myeloid leukemia (%). The orange curve indicates acute myocardial infarction without myeloid leukemia (%).

**Figure 4:**
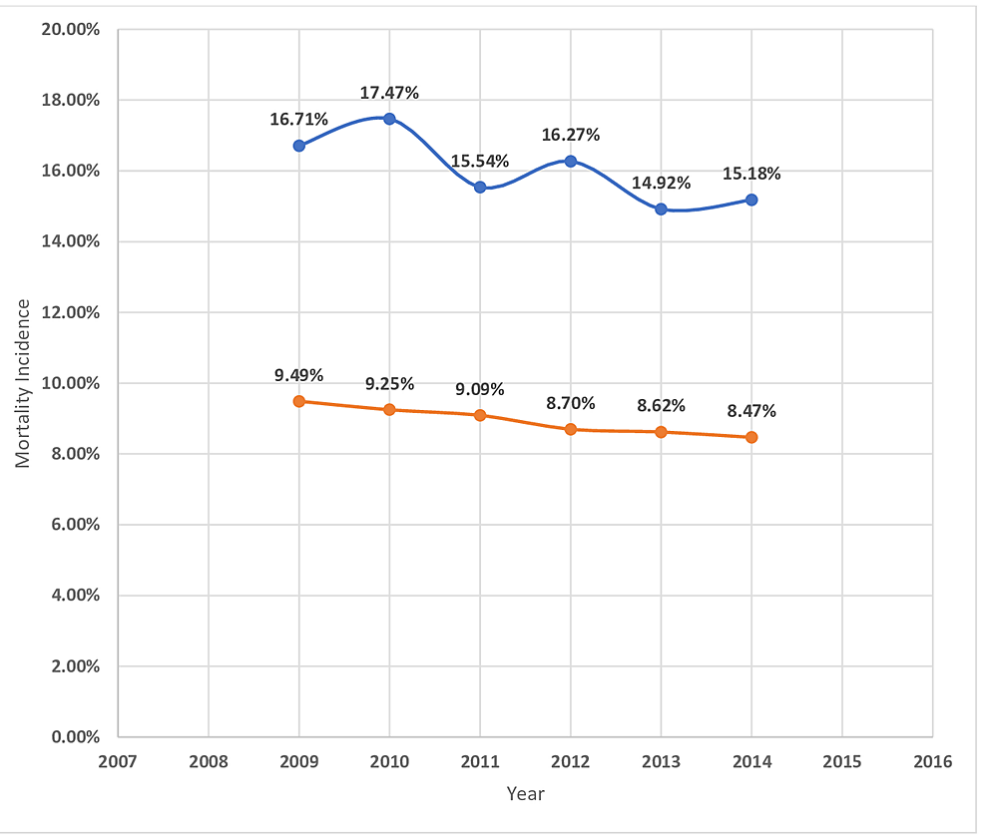
Mortality incidence in lymphocytic leukemia Trends in mortality incidence in lymphocytic leukemia with acute myocardial infarction. Blue curve indicates acute myocardial infarction with lymphocytic leukemia (%). Orange curve indicates acute myocardial infarction with lymphocytic leukemia (%).

**Figure 5:**
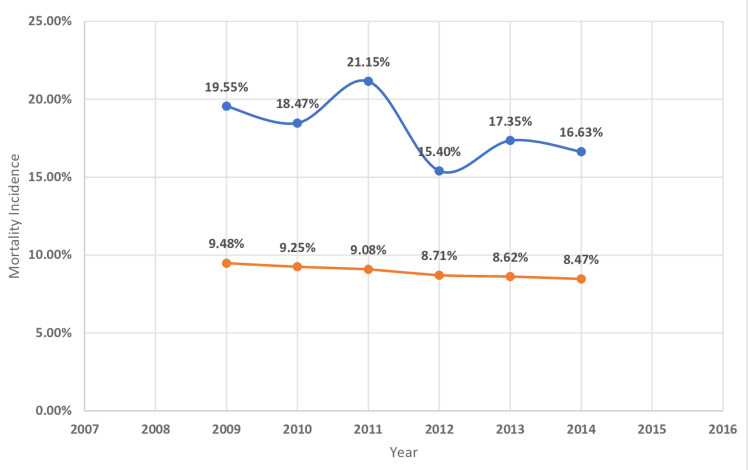
Mortality incidence in multiple myeloma. Trends in mortality incidence in multiple myeloma with acute myocardial infarction. The blue curve indicates acute myocardial infarction with underlying multiple myeloma (%). The orange curve indicates acute myocardial infarction without underlying multiple myeloma (%).

**Figure 6:**
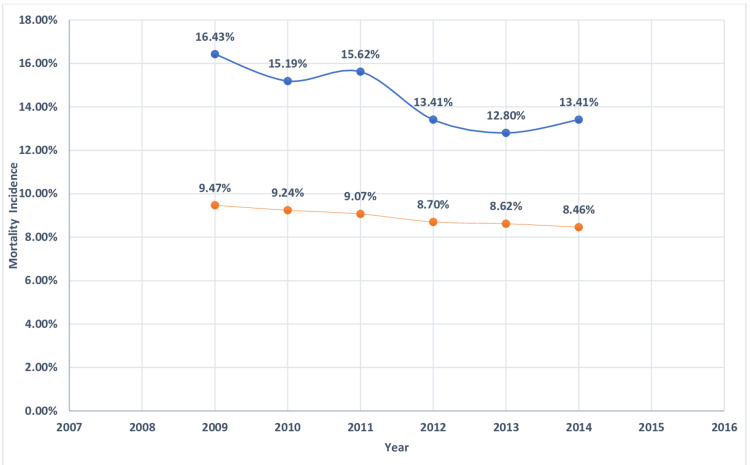
Mortality incidence in lymphoma Trends in mortality incidence in lymphoma with acute myocardial infarction. The blue curve indicates myocardial infarction in lymphoma (%). The orange curve indicates acute myocardial infarction without lymphoma (%).

**Figure 7:**
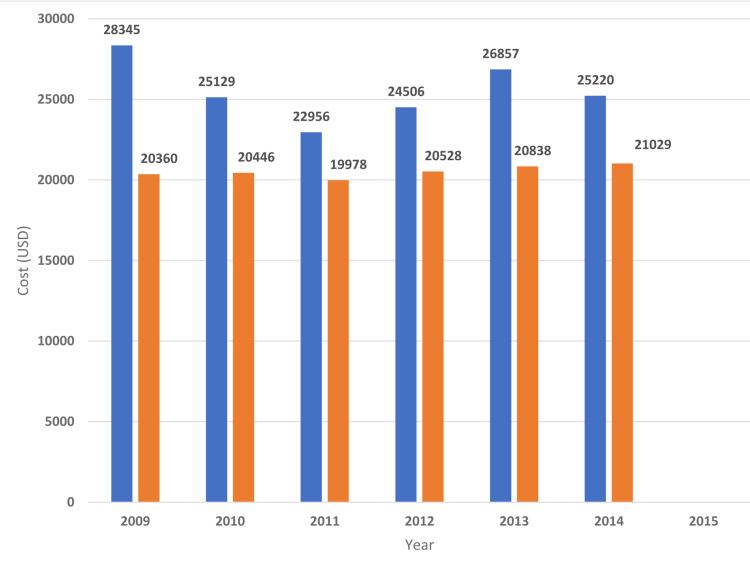
Cost of hospitalizations for hematological cancers Trends in the cost of hospitalizations for hematological cancers with myocardial infarction. Blue bars indicate acute myocardial infarction with underlying hematological neoplasms. Orange bars indicate acute myocardial infarction without underlying hematological neoplasms.

**Figure 8:**
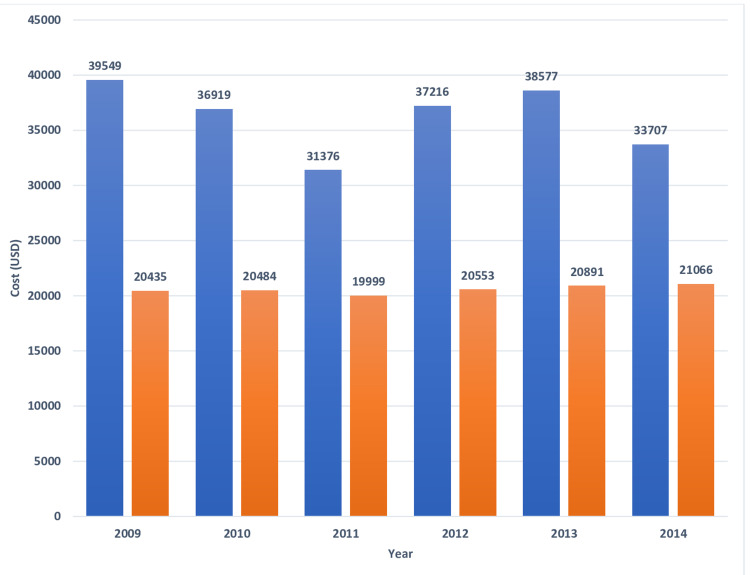
Cost of hospitalizations for myeloid leukemia Trends in the cost of hospitalizations for myeloid leukemia with myocardial infarction. Blue bars indicate acute myocardial infarction with underlying myeloid leukemia. Orange bars indicate acute myocardial infarction without underlying myeloid leukemia.

**Figure 9:**
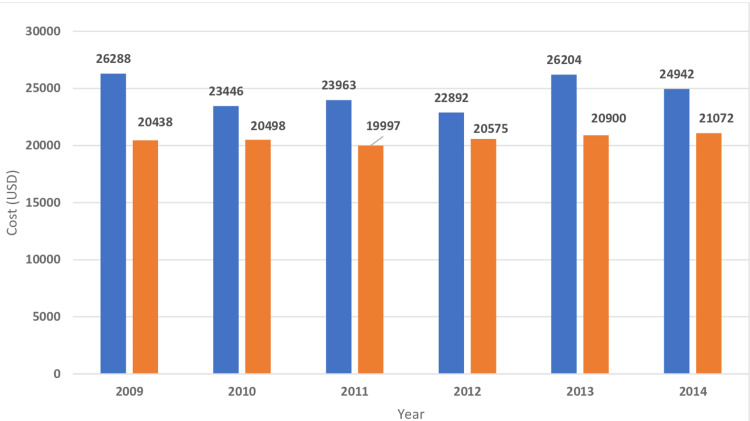
Cost of hospitalizations for lymphoma Trends in the cost of hospitalizations for lymphoma with myocardial infarction. Blue bars indicate the prevalence of myocardial infarction with underlying lymphoma. Orange bars indicate the prevalence of myocardial infarction without underlying lymphoma.

**Figure 10:**
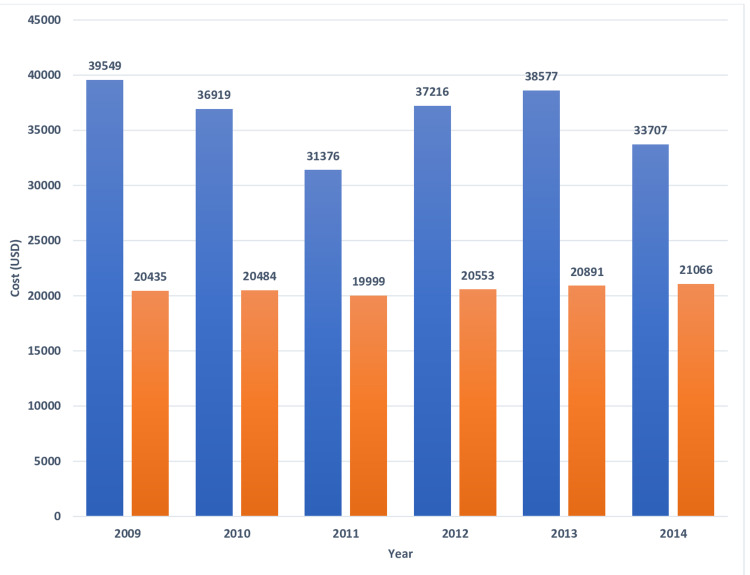
Cost of hospitalizations for multiple myeloma Trends in the cost of hospitalizations for multiple myeloma with myocardial infarction. Blue bars indicate acute myocardial infarction with underlying multiple myeloma. Orange bars indicate acute myocardial infarction without underlying multiple myeloma.

**Figure 11:**
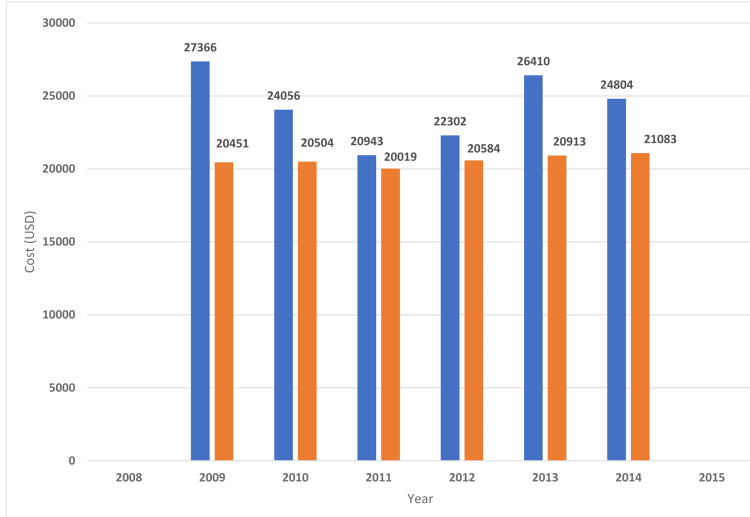
Cost of hospitalizations for lymphocytic leukemia. Trends in Cost of hospitalizations for lymphocytic leukemia with myocardial infarction. Blue bars indicate acute myocardial infarction with lymphocytic leukemia (%). Orange bars indicate acute myocardial infarction with lymphocytic leukemia (%).

The in-hospital mortality of MI was higher in patients with underlying hematological malignancy as compared to those who do not have cancer (Table 2). The highest mortality was found in myeloid leukemia (23.9%) followed by MM (17.9%), lymphocytic leukemia (15.9%), and lymphoma (14.4%) (Table 4). The length of hospital stay of MI patients with underlying hematological malignancy was higher for myeloid leukemia (11.4 ± 15.7 days) followed by MM (8.9 ± 9.5), lymphocytic leukemia( 8 ± 9), and lymphoma (7.6 ± 8.7) (Table 4). Hospitalization cost was higher in MI patients with associated with underlying myeloid leukemia($36176 ± 59713) followed by MM ($24864 ± 31725), lymphocytic leukemia ($24327 ± 34433), and lymphoma($24596 ± 33733) (Table 4). Figures 2-11 show the incidence of mortality and hospitalization cost of MI in patients with underlying hematological cancer subtypes fluctuates but decreased from 2009 compared to 2014.

## Discussion

This study evaluates the clinical outcomes of acute MI in patients with underlying hematological malignancies and their subtypes. Our study showed that acute MI in patients with hematological malignancies has higher in-hospital mortality, length of stay, and cost as compared to the patients who have MI but have no underlying cancer. Amongst the subtypes of blood neoplasms, the highest mortality, hospitalization cost, and length of stay were found in myeloid leukemia.

In our cohort of patients, the prevalence of acute MI in patients with underlying hematological malignancies was 2.4%. But the prevalence of MI in hematological malignancies varies in different studies. Park et al reported that the prevalence of MI was 1.4% in active hematological malignancies of which 78.1% were NSTEMI and 13.7% were STEMI [7]. A nationwide study performed in Sweden showed that the overall risk of CHD during the first six months after the diagnosis of cancer was 1.70% (95% CI 1.66-1.75) [8]. Navi et al. performed a retrospective matched cohort study which showed that incidence of MI was 2.0% in patients with cancer compared to 0.7% in patients without any cancer over a period of six months [9]. But in our study, the prevalence of acute MI was low in hematological cancer patients as compared to the patients without any underlying malignancy.

The risk of MI in cancer patients is determined by cancer type, stage as well as chemotherapeutic regimen. Patients with lung, gastric and pancreatic cancers had the highest rates of MI [10]. Only a few studies have been done to tell us about the prevalence of AMI in hematological cancer subtypes. A Danish population-based cohort study performed by Adelborg et al. reported that the highest risk of MI (5%) was amongst the CLL patients [11].

In general, cancer is a procoagulant state as it can activate the tissue factors and release inflammatory cytokines (i.e., TNF-α, IL-1β) which increases the risk of thrombosis and hence MI. Also, the chemotherapeutic agents used for the treatment of cancers also increase the risk of MI through different mechanisms including endothelial injury, acute arterial thrombosis, direct vasospastic effect, and plaque destabilization [11,12]. Likewise in hematological cancers, MI can occur due to thrombus formation through activation of the coagulation cascade, infiltration of the artery by leukemic cells, compression of the coronary arteries due to infiltration of the pericardium by leukemic cells, and leukostasis syndrome induced by hyperleukocytosis [13]. In most case reports the clots are aspirated instead of putting stents [13]. The reason we look at the mortality, cost, and length of stay is because the mechanism and management of the MI in hematological cancer are different than that of other non-cancer-related MI.

The 30-day incidence of mortality after arterial thromboembolic evens (arterial thromboembolic events, myocardial infarction, or stroke) was 17.6% in patients with underlying hematological malignancies vs. 11.6 % in patients without underlying hematological malignancies. In the Mayo clinic's registry of patients who underwent PCI for a STEMI, patients were found to have a three-fold higher risk of acute in-hospital and long-term non-cardiac mortality risk. However, there was no increased acute or long-term cardiac mortality risk with evidence-based cardiac treatment and care [13]. In the National Heart, Lung, and Blood Institute Dynamic Registry, a history of cancer was a significant predictor of 1-year death and MI in patients who presented with an acute MI requiring percutaneous coronary intervention (PCI) [14]. Similarly, a Dutch multicentre registry study found that patients with STEMI and cancer history had a higher all-cause and cardiac one-year mortality. Ederhy et al. noted that patients with a history of cancer did not appear to be at risk for increased in-hospital mortality among those admitted for MI [14]. For those patients with a history of cancer and acute MI, potential causes of in-hospital death may be cardiac and non-cardiac. Non-cardiac death can occur because of bleeding. The risk of bleeding events in patients with cancer is well known, as is their risk of thrombocytopenia. Cardiac death can occur due to cardiogenic shock and cardiac arrest or ventricular fibrillation. The in-hospital mortality, cost, and length of stay are higher in patients with MI and underlying hematological cancers as compared to the non-hematological cancers. The differences in the parameters are may be due to the difference in the management of acute MI. The exact cause of the highest mortality, hospitalization cost, and length of stay in myeloid leukemia is not known in our study, but it is likely due to cardiogenic shock and bleeding.

Limitations

Our study has several limitations. The nature of the database limits the ability to determine whether the patient developed MI before or after developing their respective cancers. As our study sample is large and representative of US hospitals, we demonstrated the hospital outcomes of MI with hematological cancers.

We relied on diagnosis codes for cancer subtypes and MI, which could lead to exposure and outcome misclassification; however, both ICD codes for MI and cancers are validated and used in several other studies. We did not investigate the severity and stages of cancers that could affect the development of MI. In addition, we did not study the effect of chemotherapy on the development of MI. Our study did not investigate the pathophysiology behind the higher mortality associated with cancer; however, cardiogenic shock, hypovolemic shock due to bleeding, and cardiac arrest may be implicated. Our data is from NIS, which does not show the mean duration from diagnosis/treatment to the occurrence of acute MI.

## Conclusions

Our study found that acute MI in patients with underlying hematological malignancies has higher mortality as well as increased hospitalization costs and extended length of hospital stays. In hematological cancer subtypes, the highest mortality, length of stay, and hospitalization cost were found in cases with myeloid leukemia. Future prospective studies are needed to look at in-hospital outcomes of acute MI among different hematological cancers.

## Appendices

Table 4 shows the ICD 9 codes used for the identification of cancer and acute myocardial infarction.

**Table 4:** ICD 9 codes Supplementary: ICD 9 codes used for the identification of cancer and acute myocardial infarction [15].

Single-Level Clinical Classifications Software (CCS)	Variables	ICD codes
	Acute myocardial infarction	100
	Hodgkin`s disease	37
	Non-Hodgkin`s lymphoma	38
	Multiple myeloma	40
Multi-level CSS codes		
	Myeloid leukemia	205.0
	Lymphoid leukemia	204
	Acute myocardial infarction	41000, 41001, 41002, 4101, 41010, 41011, 41012, 4102, 41020, 41021, 41022, 4103, 41030, 41031, 41032, 4104, 41040, 41041, 41042, 4105, 41050, 41051, 41052, 4106, 41060, 41061, 41062, 4107, 41070, 41071, 41072, 4108, 41080, 41081, 41082, 4109, 41090, 41091, 41092
